# *Trichonomas vaginalis* Metalloproteinase Induces Apoptosis of SiHa Cells through Disrupting the Mcl-1/Bim and Bcl-xL/Bim Complexes

**DOI:** 10.1371/journal.pone.0110659

**Published:** 2014-10-24

**Authors:** Juan-Hua Quan, Byung-Hun Kang, Guang-Ho Cha, Wei Zhou, Young-Bok Koh, Jung-Bo Yang, Heon-Jong Yoo, Min-A Lee, Jae-Sook Ryu, Heung-Tae Noh, Jaeyul Kwon, Young-Ha Lee

**Affiliations:** 1 Department of Gastroenterology, The Affiliated Hospital of Guangdong Medical College, Zhanjiang, Guangdong, China; 2 Department of Obstetrics and Gynecology, Chungnam National University Hospital, Daejeon, Korea; 3 Department of Infection Biology, Chungnam National University School of Medicine, Daejeon, Korea; 4 Department of Environmental Biology and Medical Parasitology, Hanyang University College of Medicine, Seoul, Korea; 5 Department of Medical Education, Chungnam National University School of Medicine, Daejeon, Korea; Winship Cancer Institute of Emory University, United States of America

## Abstract

To elucidate the roles of metalloproteinases and the Bcl-2 family of proteins in *Trichovaginalis. vaginalis*-induced apoptosis in human cervical cancer cells (SiHa cells) and vaginal epithelial cells (MS74 cells), SiHa cells and MS74 cells were incubated with live *T. vaginalis*, *T. vaginalis* excretory and secretory products (ESP), and *T. vaginalis* lysates, either with or without the specific metalloproteinase inhibitor 1,10-phenanthroline (1,10-PT), and examined apoptotic events and Bcl-2 signaling. The live *T. vaginalis* and the *T. vaginalis* ESP induced the release of cytochrome *c* into the cytosol, the activation of caspase-3 and caspase-9, and the cleavage of PARP. Additionally, the live *T. vaginalis*, but not the *T. vaginalis* lysate, induced the cleavage of the proapoptotic Bim protein. The live *T. vaginalis* and the *T. vaginalis* ESP, but not the *T. vaginalis* lysate, induced the dose-dependent cleavage of the antiapoptotic Bcl-xL and Mcl-1 proteins and decreased the association levels of Bcl-xL/Bim and Mcl-1/Bim complexes. We performed gelatin zymography and casein-hydrolysis assays on the live *T. vaginalis* and the *T. vaginalis* ESP to identify the apoptosis-inducing factor. Both the live *T. vaginalis* and the ESP contained high levels of metalloproteinases, of which activities were significantly inhibited by 1,10-PT treatment. Furthermore, the 1,10-PT blocked the cleavage of Bcl-xL, Mcl-1, PARP, caspase-3, and caspase-9, as well as the release of cytochrome *c* into the cytosol, and it significantly increased the association levels of the Bcl-xL/Bim and Mcl-1/Bim protein complexes, returning them to normal levels. Our results demonstrate that *T. vaginalis* induces mitochondria-dependent apoptosis in SiHa cells through the dissociation of Bcl-xL/Bim and Mcl-1/Bim complexes and that the apoptosis is blocked by the metalloproteinase inhibitor 1,10-PT. These results expand our understanding of the role of metalloproteinases in *T. vaginalis*-induced apoptosis and the signaling pathway in trichomoniasis of the cervicovaginal epithelial cells.

## Introduction

The protozoan parasite *Trichomonas vaginalis* infects the urogenital tract of humans. It is one of the most common nonviral sexually transmitted diseases [1]. Females infected with *T. vaginalis* not only develop vaginitis, but they also have an increased risk of premature delivery, low birth weight, atypical pelvic inflammatory disease, infertility, a predisposition to developing invasive cervical cancer, and an increased susceptibility to HIV infection. In males, *T. vaginalis* can cause nongonococcal urethritis and chronic prostatitis [1], [2].

Apoptosis, a highly regulated process that is essential for cell development and tissue homeostasis in eukaryotes, modulates pathogenesis in a variety of diseases [3], [4]. Mitochondria are important in the regulation and transmission of apoptotic signals and are regulated by a balance of Bcl-2-family proteins [5]. The Bcl-2 proteins are grouped into three classes based on their activities and the particular Bcl-2-homology domains they contain: antiapoptotic Bcl-2 proteins (Bcl-2, Mcl-1, and Bcl-xL), proapoptotic multidomain proteins (Bak and Bax), and BH3-only proapoptotic proteins (Bad, Bid, Puma, and Bim) [5]. The expression patterns of the proapoptotic and antiapoptotic Bcl-2 proteins regulate the mitochondrial apoptotic pathway. It is not clear, however, how mitochondrial apoptotic signaling during *T. vaginalis* infection is controlled. Live *T. vaginalis* causes neutrophilic apoptosis through the activation of caspase-3 and the reduction of Mcl-1 expression via reactive oxygen species [6], [7]. In RAW264.7 cells, *T. vaginalis* induced apoptosis through the action of Bcl-xL but not that of Bcl-2 [8]. More information is required, however, to determine the precise apoptotic-signaling pathway induced by *T. vaginalis*, especially in connection with the Bcl-2 family members.

Trichomonads secrete a number of hydrolytic enzymes [9], [10]. *T. vaginalis* proteases have been implicated as virulence factors, adherence factors, cell-detaching factors, nutrient-acquisition factors, and hemolysis factors; and they contribute to pathogenesis when released onto the host mucosal surface, helping the parasite to evade the host immune response [9]–[11]. Cysteine proteinases localized on the surface of the parasite are involved in trichomonal cytoadherence [9], and they induce apoptosis in human vaginal epithelial cells [11]. *T. vaginalis* GP63 protease, a metallopeptidase with a zinc-binding motif (HEXXH), plays a vital role in *T. vaginalis* infection process [12].

The *T. vaginalis* genome contains 13 families of metallopeptidases [13]. To elucidate whether *T. vaginalis* metalloproteinases are involved in apoptosis in human cervical cancer cell line and immortalized human vaginal epithelial cell line and to evaluate the roles of the Bcl-2 family of proteins in *T. vaginalis*-induced apoptosis, we treated SiHa cells and MS74 cells with live *T. vaginalis*, *T. vaginalis* excretory and secretory products (ESP), *T. vaginalis* lysate with or without 1,10-phenanthroline (1,10-PT). We recorded apoptotic events and Bcl-2 signaling using cell fractionation, western blotting, immunoprecipitation, gelastin zymography, and casein-hydrolysis assay. The metal ion chelator 1,10-PT can be used to inhibit zinc-dependent metalloproteases, without affecting the Ca^2+^ in the medium, as it has a much higher stability constant for Zn^2+^ than for Ca^2+^ [14], [15]. The 1,10-PT significantly inhibited metalloproteinase activity of *T. vaginalis* and parasite-induced apoptosis in SiHa cells and MS74 cells. The 1,10-PT pretreatment strongly inhibited the cleavage of PARP, caspase-3, and caspase-9 and totally blocked the release of cytochrome *c* into the cytosol. The 1,10-PT also blocked the cleavage of Bcl-xL and Mcl-1 and the degradation of Bim. Our results shed new light on the apoptosis induced by *T. vaginalis*.

## Materials and Methods

### Ethics statement

All the experimental procedures were done according to the Safety Regulation of Chungnam National University. We used cell lines from ATCC and used the established *T. vaginalis* T016 isolate and immortalized vaginal epithelial cell line MS74 cell. The *T. vaginalis* T016 isolate and MS74 cells obtained from one of the author Prof. Jae-Sook Ryu [7], [18] was kindly provided by Prof. J. K. Alderete [14], [19]. After receiving the *T. vaginalis* T016 isolate and MS74 cells from Prof. Alderete, Prof. Ryu maintained it until now. Some authors used T016 isolate and MS74 cells. Thus, this paper has one of the conditions as an exemption for the approval of the Ethics Committee of Chungnam National University.

### *T. vaginalis* cultures

The *T. vaginalis* T016 isolate obtained from one of the author Prof. Jae-Sook Ryu [7] was kindly provided by Prof. J. K. Alderete (Washington State University) [16]. Isolate T016 was cultured according to previous papers [7], [12], [16]. Briefly, *T. vaginalis* T016 isolate was cultured in glass, screw-capped tubes containing Diamond's trypticase yeast-extract maltose (TYM) medium (NAPCO, Winchester, VA, USA) supplemented with 10% heat-inactivated horse serum (Sigma-Aldrich, St Louis, MO, USA; Table 1) in 5% CO_2_ at 37°C for 24 h. The cultured parasites were monitored for motility, and their viability was determined before each experiment using trypan-blue staining (>99%).

**Table 1:** Contents of *Trichomonas vaginalis* culture media, Diamond's trypticase-yeast extract-maltose (TYM) media (pH 6.2).

Materials	Amount
Tyrypticase Peptone (BBL)	10.0 g
Yeast extract	5.0 g
Maltose monodydrate	2.5 g
L-cystein hydrochloride	0.5 g
Ascorbic acid	0.5 g
K_2_HPO_4_	0.5 g
KH_2_PO_4_	0.5 g
Penicillin-Streptomycin	3 ml
Horse serum	50 ml
Distilled water	Total 500 mL

### Preparation of *T. vaginalis* lysate and ESP

*T. vaginalis* lysate and ESP were prepared as described previously [17]. To prepare the lysate, *T. vaginalis* trophozoites were harvested in logarithmic growth phase and washed three times in PBS (pH 6.2). *T. vaginalis* pellets were resuspended in PBS, lysed by sonication, and then centrifuged at 10,000 *g* for 30 min. The supernatant was collected and stored at −70°C. To prepare the *T. vaginalis* ESP, freshly purified trophozoites (1×10^7^ cells/mL) were incubated with TYM medium at 37°C for 1 h in 5% CO_2_. After centrifugation for 30 min at 10,000 *g*, the ESP-containing supernatant was filtered through a 0.2-µm-pore filter and stored at −70°C.

The *T. vaginalis* ESP and lysate concentrations were determined by the Bradford assay with bovine serum albumin (BSA) as the standard.

### Culture of SiHa cells and MS74 cells

Human cervical cancer (SiHa) cells were obtained from the American Type Culture Collection (ATCC, Manassas, VA, USA) and maintained in Dulbecco's Modified Eagle's Medium supplemented with 10% heat-inactivated fetal bovine serum (FBS; Gibco BRL, Grand Island, NY, USA) and antibiotic–antimycotic (Gibco BRL) at 37°C in 5% CO_2_.

Immortalized human vaginal epithelial cells (MS74 cells) obtained from one of the author Prof. Jae-Sook Ryu [18] was kindly provided by Prof. J.F. Alderete (Washington State University), and grown in DMEM supplemented with 10% FBS, at 37°C, in the presence of 5% CO_2_ [19].

### Induction of apoptosis in SiHa cells and MS74 cells

In a preliminary experiment to determine the optimal *T. vaginalis*/SiHa cell ratio for inducing apoptosis, SiHa cell monolayers (1×10^6^) were washed with PBS (pH 7.4), and live *T. vaginalis* trophozoites were incubated in mixed-medium (DMEM/TYM = 2∶1) at multiplicities of infection (MOIs) of 0.5, 1, and 2 for 12, 16, and 24 h. The optimal conditions for inducing apoptosis were found to be an MOI of 2 and incubation for 16 h (Fig. S1 in File S1).

Next, 100 µg/mL *T. vaginalis* ESP or lysate was used to induce apoptosis in SiHa cells and MS74 cells. As a positive control, apoptosis was induced by treatment with staurosporine (STS, 1 µM) (Sigma-Aldrich) under identical conditions.

### DNA fragmentation analysis

DNA was isolated from 1×10^6^ SiHa cells using a genomic DNA extraction kit (iNtRON Biotechnology, Seoul, Korea). An equal amount of DNA was loaded into each well of 2% agarose gels containing ethidium bromide (0.5 µg/mL) and separated electrophoretically using Tris–borate–EDTA (pH 8.0) as the running buffer (89 mM Tris-borate, 2 mM EDTA). Migrating DNA bands were visualized with a UV transilluminator (Gel Doc, Bio-Rad Laboratories Ltd, Hercules, CA, USA).

### Cytosol fractionation

Cytosolic extracts free of nuclei and mitochondria were prepared as described previously [20]. Briefly, cells were washed in ice-cold PBS (pH 7.2) and then in a hypotonic extraction buffer (HEB; 50 mM PIPES, 50 mM KCl, 5 mM EGTA, 2 mM MgCl_2_, 1 mM dithiothreitol, and 0.1 mM PMSF; pH 7.4) before being harvested by centrifugation. The pellets were resuspended in HEB and lysed in a Dounce homogenizer. The cell lysates were then centrifuged at 100,000 *g* for 60 min at 4°C, and the supernatants were flash-frozen in cold ethanol, aliquoted, and stored at −80°C.

### Western blotting

SiHa cells or MS74 cells were incubated with live *T. vaginalis, T. vaginalis* ESP, *T. vaginalis* lysate, or STS for 16 h and then harvested. After the cells were washed in PBS, the proteins were extracted using the PRO-PREP Protein Extraction Solution (iNtRON Biotechnology) and supplemented with a complete cocktail of protease inhibitors (Roche, Basel, Switzerland) for 15 min on ice. After centrifugation at 14,000 *g* for 15 min at 4°C, the supernatant was collected, and equal amounts of proteins from each sample were separated by SDS-PAGE and transferred to a polyvinylidene difluoride membrane. The membranes were blocked in Tris-buffered saline (20 mM Tris, 137 mM NaCl; pH 7.6) containing 0.1% Tween-20 (TBST) and 5% skim milk. After being washed once in TBST, the membranes were incubated overnight at 4°C with the primary antibodies diluted in TBST supplemented with 5% BSA. The antibodies were: anti-cytochrome *c*, anti-cleaved caspase-9, anti-cleaved caspase-3, anti-cleaved caspase-8, anti-poly-(ADP-ribose) polymerase (PARP), anti-Bcl-2, anti-Bcl-xL, anti-Mcl-1, anti-Bim, anti-Bax, anti-Bid, anti-Bak, anti-Puma (all from Cell Signaling Technology Inc., Beverly, MA, USA), anti-α tubulin, and anti-Bcl-xL (Santa Cruz Biotechnology, Santa Cruz, CA, USA). Following three consecutive washes in TBST, the membranes were incubated for 90 min with horseradish peroxidase-conjugated anti-mouse or anti-rabbit IgG (Santa Cruz Biotechnology) diluted 1∶10,000 with incubation buffer, as described above. After extensive washing, the bound secondary antibodies were visualized using an enhanced ECL chemiluminescence detection kit (GE Healthcare, Little Chalfont, UK).

### Gelatin zymography

The proteolytic activities of the live *T. vaginalis* and the *T. vaginalis* ESP, each with or without the specific metalloproteinase inhibitor 1,10-PT, were assayed by 10% SDS-PAGE with 0.1% (w/v) gelatin incorporated into the gel as a substrate. For the preparation of the 1,10-PT-pretreated *T. vaginalis* ESP, fresh *T. vaginalis* trophozoites were treated for 30 min with 5 mM 1,10-PT, washed three times with PBS, and then incubated by the same methods described for the preparation of the *T. vaginalis* ESP. Finally, the 1,10-PT-pretreated *T. vaginalis* ESP was collected from the supernatants. The viability of the *T. vaginalis* was not affected by incubation with 5 mM 1,10-PT for 30 min (Fig. S2 in File S1).

After electrophoresis, the SDS was removed by incubation with 2.5% Triton X-100 for 1 h at room temperature. The gel was then equilibrated with Zymogram developing buffer (50 mM Tris base, 0.2 M NaCl, 5 mM CaCl_2_, and 0.02% Brij 35) for 30 min with gentle agitation. Fresh Zymogram developing buffer was then added, and the gel was incubated at 37°C for 18 h to promote proteolysis. The gel was stained for 30 min with 0.5% (w/v) Coomassie brilliant blue R-250 and destained with destaining solution containing methanol–acetic-acid–water (50∶10∶40). Protease activity was detectable as a clear zone against the blue background.

### Casein-hydrolysis assay

The protease activity of *T. vaginalis* was measured by a casein-hydrolysis assay using a protease assay kit (Pierce Co., Rockford, IL USA). All assays were performed in microtiter plates. Briefly, a solution of 200 µg succinylated casein in a 100 µL volume (prepared in 50 mM borate, pH 8.5, at a concentration of 2 mg/mL) was added to the wells on the left half of the plate, and an equal volume (100 µL) of the buffer was added to the wells on the right half of the plate. Fifty microliters of each sample was added to both the succinylated casein wells and the corresponding blank wells. The samples were incubated for 20 min at 37°C, and then 50 µL diluted (1∶149) trinitrobenzenesulfonic acid (TNBA) was added to each well and incubated for 20 min at room temperature. Color development was measured at a wavelength of 450 nm using a Tecan Sunrise Reader (Tecan Austria GmbH, Groedig, Austria).

### Immunoprecipitation

SiHa cells or MS74 cells stimulated with live *T. vaginalis, T. vaginalis* ESP, *T. vaginalis* lysate, or STS were washed twice in PBS and then resuspended in RIPA buffer (Thermo Scientific, Rockford, IL, USA) containing a cocktail of protease inhibitors (Roche), and the cells were subsequently disrupted by repeated aspiration through a 21-gauge needle. Cellular debris was removed by centrifugation at 10,000 *g* for 10 min at 4°C. To preclear the lysate, the supernatant was mixed with protein A/G plus-agarose (Santa Cruz Biotechnology, Santa Cruz, CA, USA) and incubated at 4°C for 30 min with rocking. After centrifugation at 2,500 *g* for 5 min at 4°C, the supernatants were incubated with anti-Bim and anti-Bcl-xL (1∶50 and 1∶100 dilutions, respectively) for 2 h at 4°C with rocking, and then protein A/G plus-agarose was added. After overnight incubation, the samples were centrifuged at 2,500 *g* for 5 min at 4°C. The pellets were then washed four times with RIPA buffer and prepared for western blotting.

### Statistical analysis

The data are presented as means ± standard deviations. Statistical significance was determined by ANOVA using of SPSS 16.0 software (Chicago, Illinois, USA). All experiments were performed at least in triplicate on separate days. Differences were considered significant at *P*-values<0.05.

## Results

### Live *T. vaginalis* and *T. vaginalis* ESP induced mitochondria-dependent apoptosis in SiHa cells

**Figure 1:**
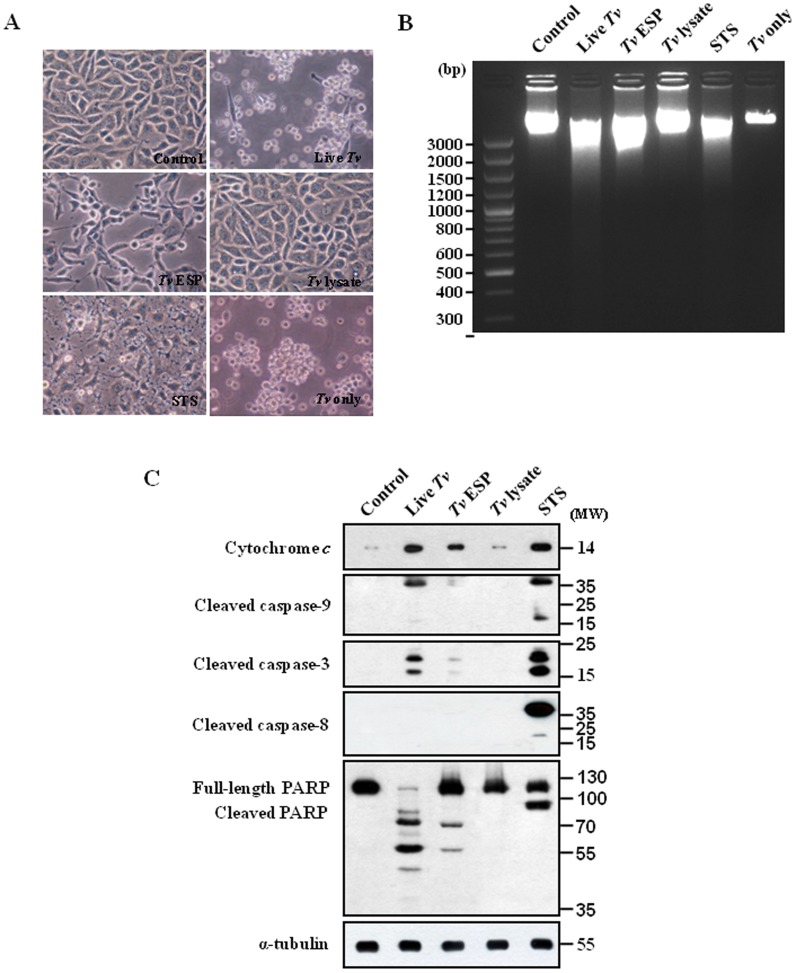
Mitochondria-dependent apoptosis in SiHa cells after treatment with *Trichomonas vaginalis* antigens. (A) Micrographs of SiHa cells incubated with live *T. vaginalis* (MOI = 2), *T. vaginalis* excretory and secretory products (ESP) (100 µg/mL), *T. vaginalis* lysate (100 µg/mL), or STS (1 µM) for 16 h. (B) DNA fragmentations of the SiHa cells were determined by agarose-gel electrophoresis. (C) The cytosolic fraction (only for dectection of cytochrome *c*) or protein extracts (detection for the others beside of cytochrome *c*) of the SiHa cells were subjected to western blotting using the indicated antibodies. A representative image from three independent replicates is shown.

As *T. vaginalis* binds to human host epithelial cells to establish and maintain an infection and in women the parasite resides in the vagina and colonizes the cervix, we chose SiHa cells, the carcinorma cell line of the cervix as in vitro experimental model. To study whether and how *T. vaginalis* induces cell death in SiHa cells, the cells were incubated with live *T. vaginalis*, *T. vaginalis* ESP, *T. vaginalis* lysate, or staurosporine (STS) (1 µM) for 16 h (Fig. 1A). The ESP was prepared from the culture medium of trophozites and the lysates was the soluble fraction of the sonicated trichomonads. Treatment of staurosporine, a well-known inducer of apoptosis in a wide range of cell lines, generated pronounced cell debris and changes in morphology, such as cell lysis, loss of spindle shape and sometimes detachment from the bottom. The decreases in cell number and slenderness in cell morphology were apparent in the SiHa cells treated with live *T. vaginalis*. Similarly, *T. vaginalis* ESP also induced cell death in the SiHa cells, even though it is less than that by live *T. vaginalis* treatment. However, the *T. vaginalis* lysate-treated cells looked similar to healthy control cells without any treatment (Fig. 1A). Live *T. vaginalis*, *T. vaginalis* ESP, and STS in SiHa cells induced nucleosomal DNA fragmentation, one of the most prominent features of apoptosis, but *T. vaginalis* lysate did not (Fig. 1B). The results suggest that live *T. vaginalis* induced cell death in the SiHa cells and ESP from *T. vaginalis* produced the similar effects on the cells.

As apoptosis involves significant morphological changes induced by caspases, which are activated upon induction of apoptotic signaling and cleave downstream substrate molecules to facilitate the apoptotic cascade, we examined the activation of various caspases in the SiHa cells using antibodies that specifically recognizes the cleaved form of caspase-3, -8, or -9 (Fig. 1C). As shown in Fig. 1C, the 17- and 19-kDa forms of cleaved caspase-3 were strongly observed in the cells treated with live *T. vaginalis,* like in the staurosporine-treated cells. The functional activity of the activated caspase-3 was examined against a caspase-3 substrate PARP. Staurosporine-treated SiHa cells produced a strong cleaved form p89 of PARP. In the SiHa cells treated with live *T. vaginalis* for 16 h, the original 113 kDa form of PARP was almost not detected and several forms of cleaved PARP were detected, indicating that another pathway is also involved on the cleavage of PARP. Treatment of *T. vaginalis* ESP resulted in the cleavage of caspase-3 and PARP, which was similar to but weaker than the one by live *T. vaginalis*.

As markers of the intrinsic apoptotic pathway which involves signaling through the mitochondria, we examined release of cytochrome *c* from the mitochondria and subsequent caspase-9 cleavage. We observed cleaved forms of caspase-9, p37 and p17, and also strong cytosolic cytochrome *c*, which were also detected in staurosporine-treated SiHa cells (Fig. 1C). As indicators of the involvement of the extrinsic apoptotic pathway through activation of cell surface death receptors, cleaved forms of caspase-8, p43 and p18, were detected in staurosporine-treated cells. However, we could not detect any cleaved forms of caspase-8 in live *T. vaginalis*-treated cells, suggesting that activation of cell surface death receptors may not be involved in *T. vaginalis*-induced apoptotic process. In *T. vaginalis* lysates-treated cells there was no sign of apoptotic process (Fig. 1C). All these results suggest that in *T. vaginalis*-induced apoptotic process there was a strong involvement of signaling through the mitochondria such as release of cytochrome *c* from the mitochondria, subsequent caspase-9 cleavage, and activation of caspase-3.

### 1,10-PT inhibited metalloproteinase activity of *T. vaginalis* and parasite-induced apoptosis in SiHa cells

**Figure 2:**
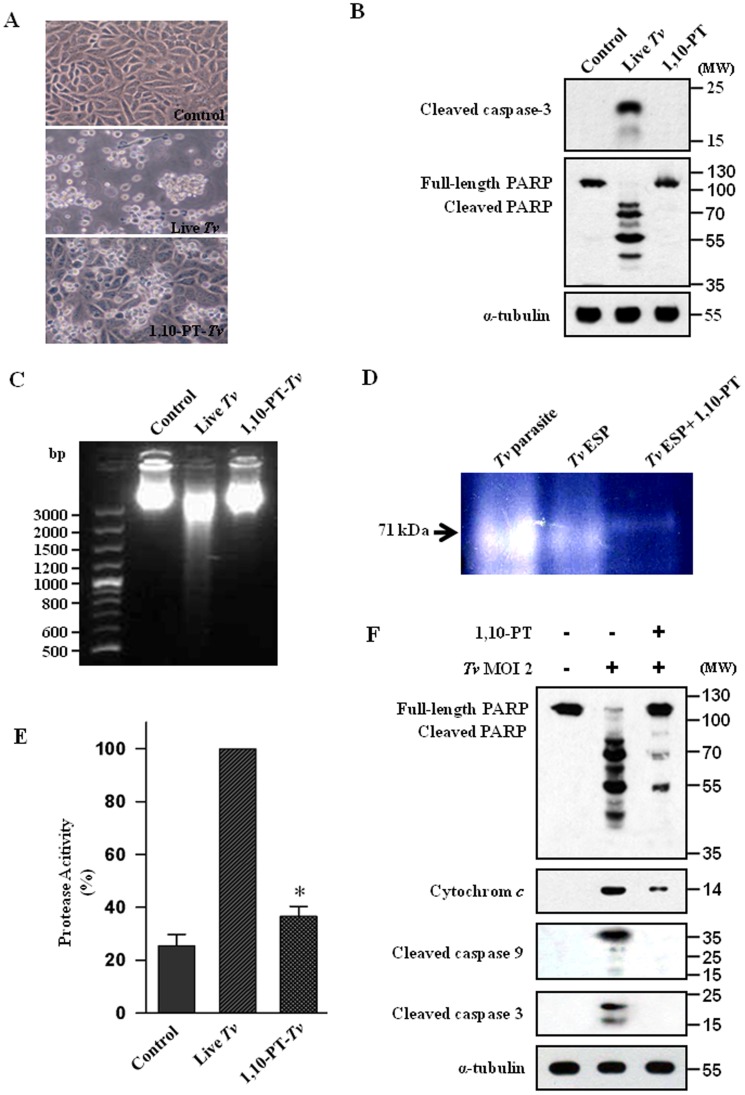
Effects of 1,10-phenanthroline on metalloproteinase activity and the inhibition of *T. vaginalis*-induced apoptosis. (A) Micrographs of SiHa cells incubated with live *T. vaginalis* or with metalloproteinase inhibitor 1,10-phenanthroline (1,10-PT, 5 mM) pretreated *T. vaginalis* for 16 h. (B) SiHa cells were treated with live *T. vaginalis* or incubated with 1,10-PT for 16 h and then the protein extracts were analyzed by western blotting using indicated antibodies. (C) SiHa cells incubated with live *T. vaginalis* (MOI = 2) or, 1,10-PT-pretreated *T. vaginalis* for 16 h. The cells were collected, and DNA fragmentation was determined by agarose-gel electrophoresis. (D) Live *T. vaginalis*, *T. vaginalis* ESP and 1,10-PT-pretreated *T. vaginalis* ESP were subjected to electrophoresis. Substrate proteolytic activity was determined by 10% SDS-PAGE with 0.1% gelatin. (E) SiHa cells were treated as in Fig. 2C, the supernatants were collected and then the protease activities were determined by casein-hydrolysis assay. Each assay was carried out in triplicate, and the results shown are the relative percentages of protease activities in the cells infected with the pretreated parasites compared with those in the *T. vaginalis*-infected cells, which were set as 100%. * *P*<0.05. (F) The cytosolic fraction (only for dectection of cytochrome *c*) or protein extracts (detection for the others beside of cytochrome *c*) of the SiHa cells were isolated, and western blotting was performed with using indicated antibodies. A representative result of three independent replicates is shown.

The possible involvement of metalloproteases in *T. vaginalis*-induced cell death was evaluated by assessing the inhibitory effect of 1,10-PT. We tested different concentrations of 1,10-PT (ranging from 0.5 to 10 mM) to find out the appropriate concentration to be used without killing the parasites (Fig. S2 in File S1). We found that pretreatment of 5 mM 1,10-PT to *T. vaginalis* could prevent the *T. vaginalis*-induced cell death of SiHa cells to a significant degree (Fig. 2A). Pretreatment of 1,10-PT to *T. vaginalis* inhibited cleavages of caspase-3 and PARP (Fig. 2B) and DNA fragmentation (Fig. 2C) in the SiHa cells induced by the parasite infection. Our date suggest that zinc-dependent metalloproteases in *T. vaginalis* is involved in the cell death of SiHa cells infected with *T. vaginalis*.

In order to assess the role of metalloproteases in *T. vaginalis*-induced SiHa cell death, the cell-associated and extracellularly secreted peptidases of *T. vaginalis* as a form of live *T. vaginalis* and *T. vaginalis* ESP were analyzed by measuring peptidase activity in gelatin-containing zymograms (Fig. 2D). Live *T. vaginalis* and *T. vaginalis* ESP showed a strong band of gelatinase activity around 71 kDa, which was almost completely inhibited by 1,10-PT, suggesting that *T. vaginalis* has the cell-associated metalloproteases as well as the extracellularly released metalloproteases.

To examine whether the SiHa cells infected with live *T. vaginalis* have an enhanced protease activity, the protease activity in the supernatants of the SiHa cells was determined by the casein-hydrolysis assay (Fig. 2E). The SiHa cells infected with live *T. vaginalis* showed much higher protease activity than the untreated cells. The protease activity in the SiHa cells infected with the 1,10-PT-pretreated *T. vaginalis* was strongly diminished compared with that in the SiHa cells infected with live *T. vaginalis*. These data suggest that the metalloprotease activity of *T. vaginalis* is important for enhanced protease activity in the SiHa cells infected with live *T. vaginalis*.

To examine how much caspase-3 activity is contributing to the enhanced protease activity in the live *T. vaginalis*-infected SiHa cells, we added a specific caspase-3 inhibitor Z-DEVD-FMK into SiHa cells and determined the protease activity in the supernatants of the SiHa cells through the casein-hydrolysis assay (Fig. S3 in File S1). The caspase-3 inhibitor Z-DEVD-FMK significantly inhibited the protease activity in the supernatants of the SiHa cells. Inhibitory effect of 1,10-PT was much stronger than that of Z-DEVD-FMK, suggesting that when the SiHa cells were infected by *T. vaginalis* there is strong enhancement of metalloprotease activity in the SiHa cells (Fig. S4 in File S1).

To probe the role of metalloproteases of *T. vaginalis* how affect the apoptosis-relevant molecules of the SiHa cells, we pretreated the *T. vaginalis* with or without 1,10-PT and then analyzed the cytosolic fraction of the infected SiHa cells (Fig. 2F). The indicators of mitochondria-dependent apoptotic pathway, such as cytosolic cytochrome *c*, cleaved forms of caspase-9 and -3, were markedly inhibited by pretreatment of *T. vaginalis* with 1,10-PT. The cleavage of PARP was also strongly inhibited by 1,10-PT pretreatment.

### *T. vaginalis* infection induced the early cleavage of Bcl-xL and Mcl-1 in a MOI-dependent manner

**Figure 3:**
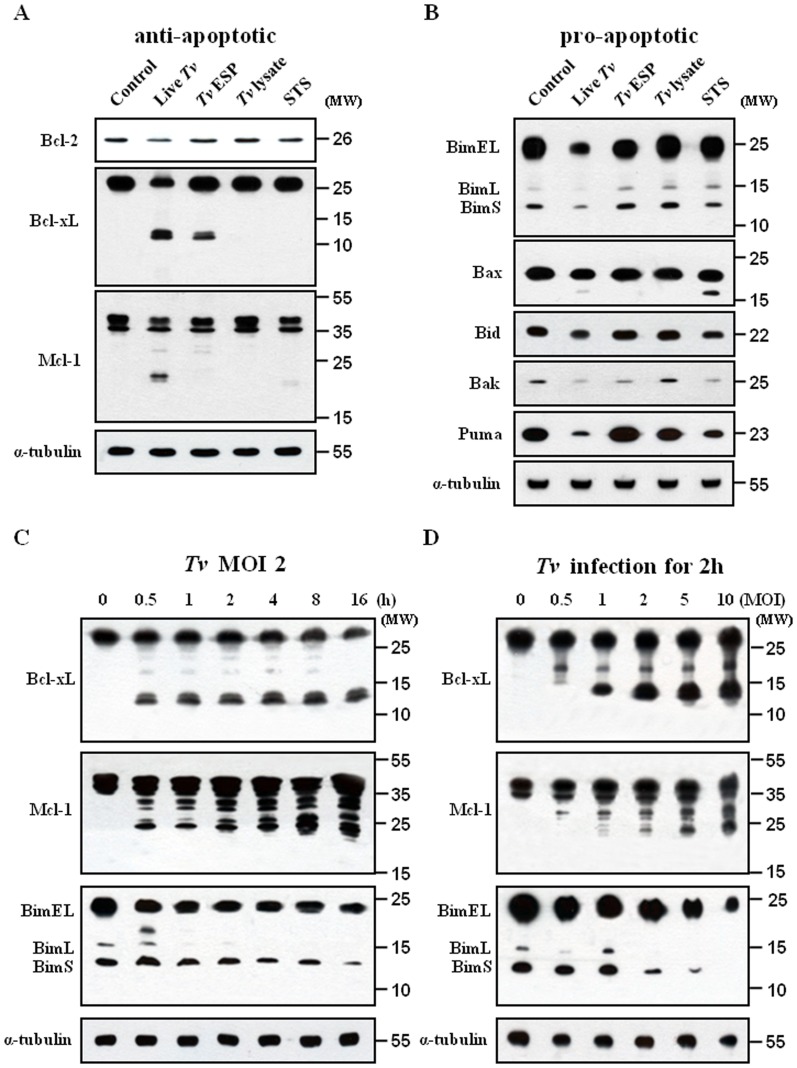
Western blot analysis of Bcl-2 family proteins in SiHa cells treated various *T. vaginalis* antigens. SiHa cells were treated as in Fig. 1A, the protein extracts were analysed with (A) anti-Bcl-2, anti-Bcl-xL, anti-Mcl-1, and anti-α tubulin antibodies or (B) anti-Bim, anti-Bax, anti-Bid, anti-Bak, and anti-Puma antibodies. SiHa cells were stimulated with *T. vaginalis* (MOI = 2) for the indicated times (C) or at the indicated MOI for 2 h (D). The protein extracts were analyzed by western blotting using anti-Bcl-xL, anti-Mcl-1 and anti-Bim antibodies. A representative result of three independent replicates is shown.

As the Bcl-2 family members are major regulators of mitochondrial integrity and mitochondria-dependent caspase activation, we examined the protein expression of anti-apoptotic (Bcl-2, Bcl-xL, and Mcl-1) and pro-apoptotic (Bim, Bax, Bid, Bak, and Puma) proteins by western blotting. Bcl-2 protein expression in the SiHa cells decreased after treatment with live *T. vaginalis* for 16 h (Fig. 3A).Treatment of live *T. vaginalis* induced a significant cleavage of Bcl-xL and Mcl-1 protein. The staurosporine treatment induced a slight decrease of the protein expression of Bcl-2 and a small amount of cleaved forms of Mcl-1. However, no changes in those proteins were detected in the SiHa cells treated with *T. vaginalis* lysate. The expression of the pro-apoptotic proteins such as Bim, Bax, Bid, Bak, Bax and Puma was significantly reduced in the cells treated with live *T. vaginalis* for 16 h (Fig. 3B). *T. vaginalis* ESP induced a small decrease of Bak. The staurosporine treatment reduced the protein amount of Puma, Bak, Bid and Bax. *T. vaginalis* lysate did not induce any change in the protein expression levels of the pro-apoptotic proteins. These results demonstrate that treatment of *T. vaginalis* to the SiHa cells for 16 h could selectively target a subset of the Bcl-2 family proteins for cleavage or degradation.

As the treatment of live *T. vaginalis* for 16 h induced characteristically the strong cleaved forms of anti-apoptotic Bcl-xL and Mcl-1, we examined the kinetics of live *T. vaginalis*-induced cleavage of them in the SiHa cells during the shorter time. Treatment of live *T. vaginalis* at MOI 2 induced the rapid cleavage of Mcl-1 and Bcl-xL in the SiHa cells, which was detectable as soon as 30 min after the live *T. vaginalis* infection (Fig. 3C). The intensity of the cleaved forms of Mcl-1 and Bcl-xL increased very slowly along the incubation period. Immunoblot analysis detected the co-presence of three Bim isoforms [Extra-Long (EL), Long (L), and Short (S)]. The protein amount of pro-apoptotic Bim, which is known to interact with Bcl-xL and Mcl-1, got decreased slightly along the time course of the treatment. At 16 h, Bcl-xL and Mcl-1 produced strong cleaved forms and the amount of Bim was reduced as seen in Fig. 3C. These data suggest that live *T. vaginalis* produced a strong cleavage of anti-apoptotic Bcl-xL and Mcl-1 in the SiHa cells at early time, even 30 min, of the infection.

Furthermore, infection of the SiHa cells with *T. vaginalis* resulted in a MOI-dependent cleavage of Bcl-xL and Mcl-1 in the SiHa cells (Fig. 3D). At MOI 1, all cleaved forms of Bcl-xL were detected. In the case of Mcl-1, MOI 2 generated most of the cleaved forms. We could observe the MOI-dependent decrease of BimEL, BimL, and BimS, markedly from MOI 2 to MOI 10. Our data suggest that the cleavage of Bcl-xL and Mcl-1 was increased incrementally with the parasite burden.

### 1,10-PT inhibited the cleavage of Bcl-xL and Mcl-1

**Figure 4:**
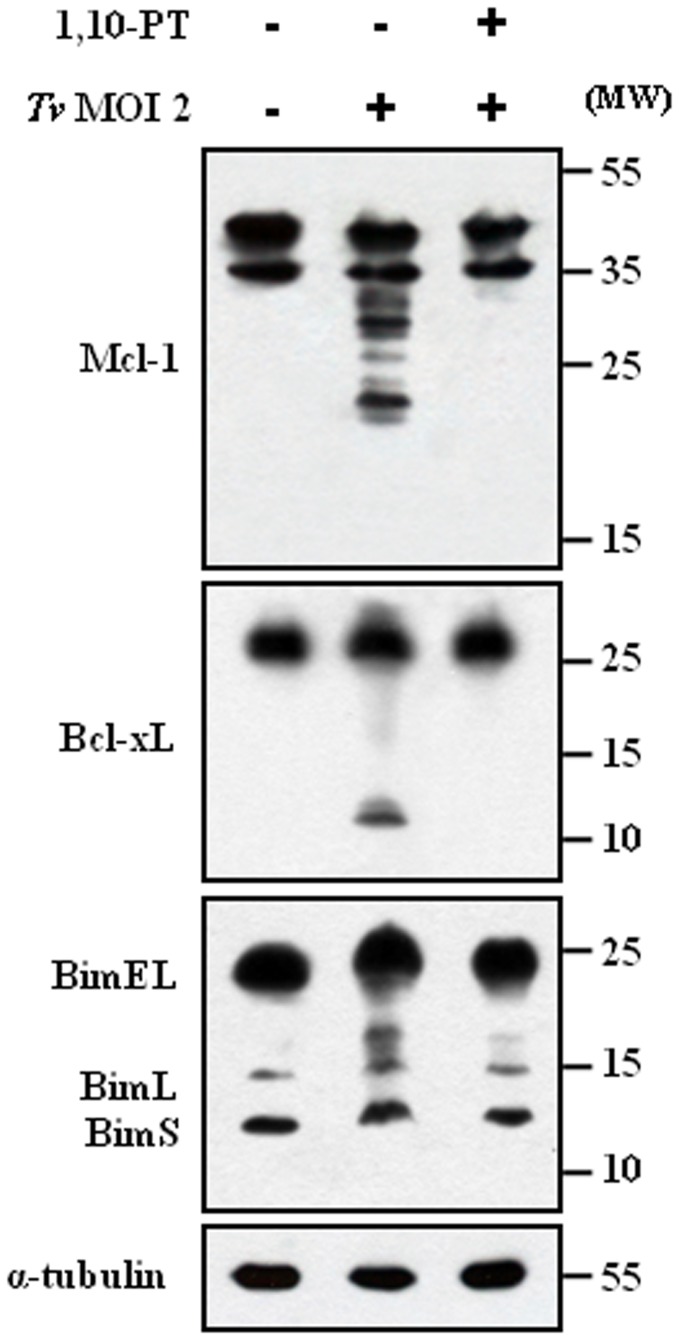
Effects of 1,10-PT on the cleavage of Mcl-1, Bcl-xL, and Bim in SiHa cells. SiHa cells were incubated with *T. vaginalis* or 1,10-PT-pretreated *T. vaginalis* for 16 h. The protein extracts were anlyzed by western blotting using the indicated antibodies. A representative result of three independent replicates is shown.

As the indicators of mitochondria-dependent apoptotic pathway, such as cytosolic cytochrome *c*, cleaved forms of caspase-9 and -3, were markedly inhibited by pretreatment of *T. vaginalis* with the Zn-containing metalloprotease inhibitor 1,10-PT, we reasoned the effects of 1,10-PT on the Bcl-2 family members as major regulators of mitochondria-dependent caspase activation. Here, we examined its effects on a cleavage of anti-apoptotic Bcl-xL and Mcl-1, which was a strong feature induced by *T. vaginalis* infection. For the experiment, SiHa cells were infected with *T. vaginalis*, untreated or pretreated with 1,10-PT, at an MOI 2 for 2 h. Preincubation of *T. vaginalis* with 1,10-PT abolished the characteristic cleavage of Mcl-1 and Bcl-xL in the SiHa cells following live *T. vaginalis* (Fig. 4), strongly suggesting that metalloproteases in *T. vaginalis* are responsible for the cleavage of Mcl-1 and Bcl-xL in the apoptotic SiHa cells.

We examined the effects of the caspase-3 inhibitor Z-VEVD-FMK on the cleavage of Mcl-1 and Bcl-xL in the apoptotic SiHa cells, because the cleavage of Mcl-1 and Bcl-xL by caspase-3 was proposed as a mechanism to enhance and accelerate the mitochondria-dependent apoptosis [21], [22]. The metallorpotease inhibitor 1,10-PT blocked completely the cleavage of Mcl-1 and Bcl-xL. The Bcl-xL cleavage was completely inhibited by the caspase-3 inhibitor. However, the caspase-3 inhibitor was able to partially inhibit the cleavage of Mcl-1. The metallorpotease inhibitor could almost block the cleavage of PARP. But the PARP cleavage was slightly inhibited by the caspase-3 inhibitor (Fig. S4 in File S1).

### 1,10-PT inhibited the dissociation of the Bcl-xL/Bim and Mcl-1/Bim complexes

**Figure 5:**
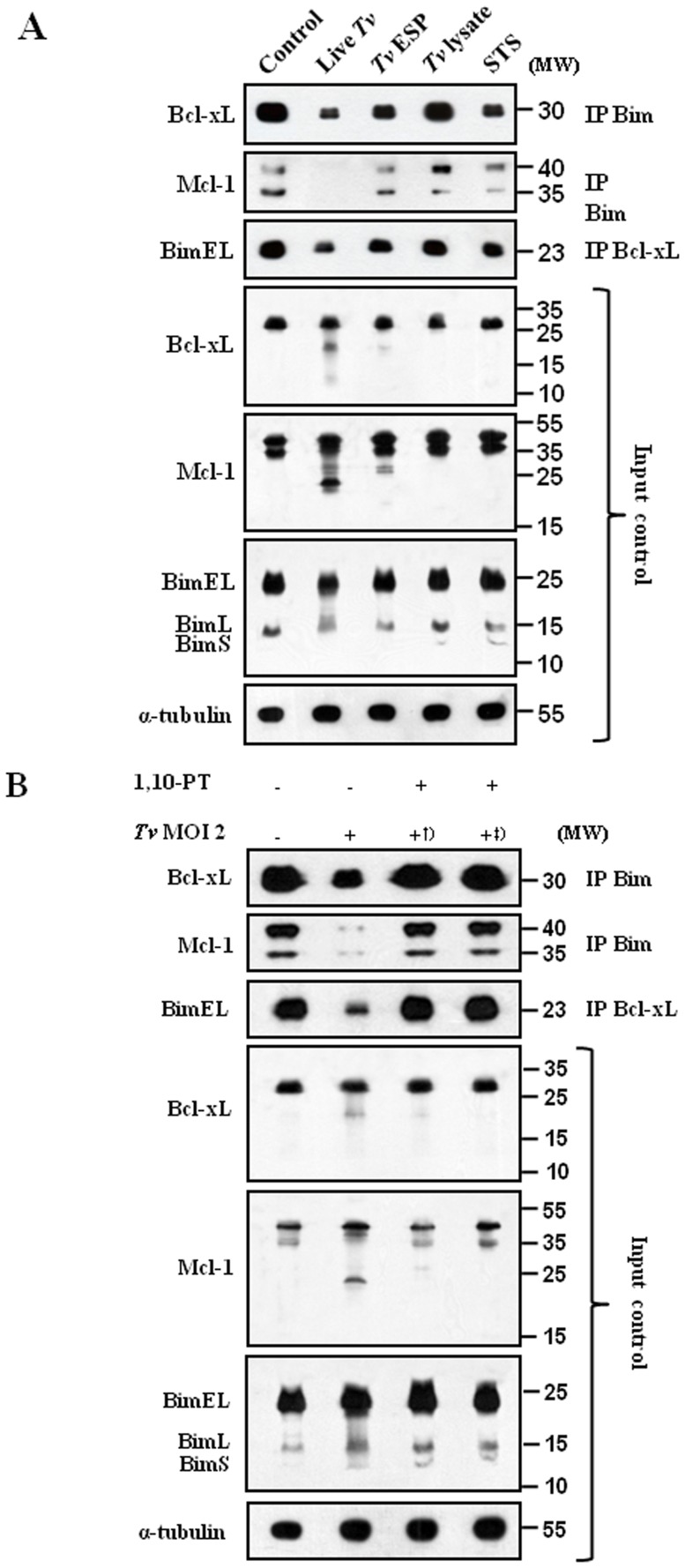
1,10-PT suppressed the *T. vaginalis*-induced dissociation of the Bcl-xL/Bim and Mcl-1/Bim complexes in SiHa cells. SiHa cells were treated as in Fig. 1A (A) or incubated with *T. vaginalis*, 1,10-PT-pretreated *T. vaginalis* ^†^, or *T. vaginalis* and 1,10-PT simultaneously^‡^ (B). Whole-cell lysates were subjected to immunoprecipitation (IP) using anti-Bim and anti-Bcl-xL antibodies. IP and input samples were resolved by SDS-PAGE and probed with the indicated antibodies. A total of 1% of the cell extract volume from each sample was used as input control.

The critical function of anti-apoptotic Bcl-xL and Mcl-1 in preventing apoptosis has been proposed to sequester BH3-only molecules into stable complexes, thus preventing the activation of Bax and Bak, and also a BH3-only molecule Bim was shown to directly activate Bax and Bak to release cytochrome *c* [23]. Based on this model that anti-apoptotic Bcl-xL and Mcl-1 inhibit apoptosis by sequestering BH3-only molecules including Bim, we examined the cleavage of Bcl-xL and Mcl-1 and the disruption of Bcl-xL/Bim and Mcl-1/Bim complexes following *T. vaginalis* infection (Fig. 5A). Bim was immunoprecipitated to be tested for the association with Bcl-xL and Mcl-1. Bcl-xL and Mcl-1 were tightly associated with Bim in the cells without any treatment. The association of Bcl-xL and Mcl-1 with Bim was strongly decreased in cells treated with live *T. vaginalis*. Treatment of *T. vaginalis* ESP or staurosporine induced a small decrease in this association. The association levels in the cells treated with *T. vaginalis* lysate was similar to those in the control cells. When Bcl-xL was immunoprecipitated and the associated BimEL was examined, the association of BimEL with Bcl-xL was strongly decreased in the cells treated with live *T. vaginalis*. These results suggest that live *T. vaginalis* infection disrupt Bcl-xL/Bim and Mcl-1/Bim complexes and release Bim likely to activate the mitochondria-dependent apoptotic pathway.

As the 1,10-PT-inhibitable metalloproteases in *T. vaginalis* are responsible for the cleavage of Mcl-1 and Bcl-xL in the apoptotic SiHa cells, we examined the effects of 1,10-PT on the disruption of Bcl-xL/Bim and Mcl-1/Bim complexes (Fig. 5B). We took two ways of 1,10-PT treatment. First, *T. vaginalis* were pre-treated with 1,10-PT for 30 min and then washed. Second, when SiHa cells were incubated with *T. vaginalis*, 1,10-PT was added to the incubation medium together with the parasites. No matter which mode of treatment, treatment of 1,10-PT prevents the disruption of Bcl-xL/Bim and Mcl-1/Bim complexes in *T. vaginalis*-infected SiHa cells.

### *Trichonomas vaginalis* metalloproteinase induces apoptosis of MS74 cells through disrupting the Mcl-1/Bim and Bcl-xL/Bim complexes

As we observed the critical role of metalloproteases in *T. vaginalis*-induced cell death of the human cervical cancer cell line SiHa cells, we also chose immortalized human vaginal epithelial cell line MS74 cells as another in vitro experimental model. It was well known that *T. vaginalis* binds to human host epithelial cells to establish and maintain an infection and especially in women the parasite resides in the vagina and colonizes the cervix.

**Figure 6:**
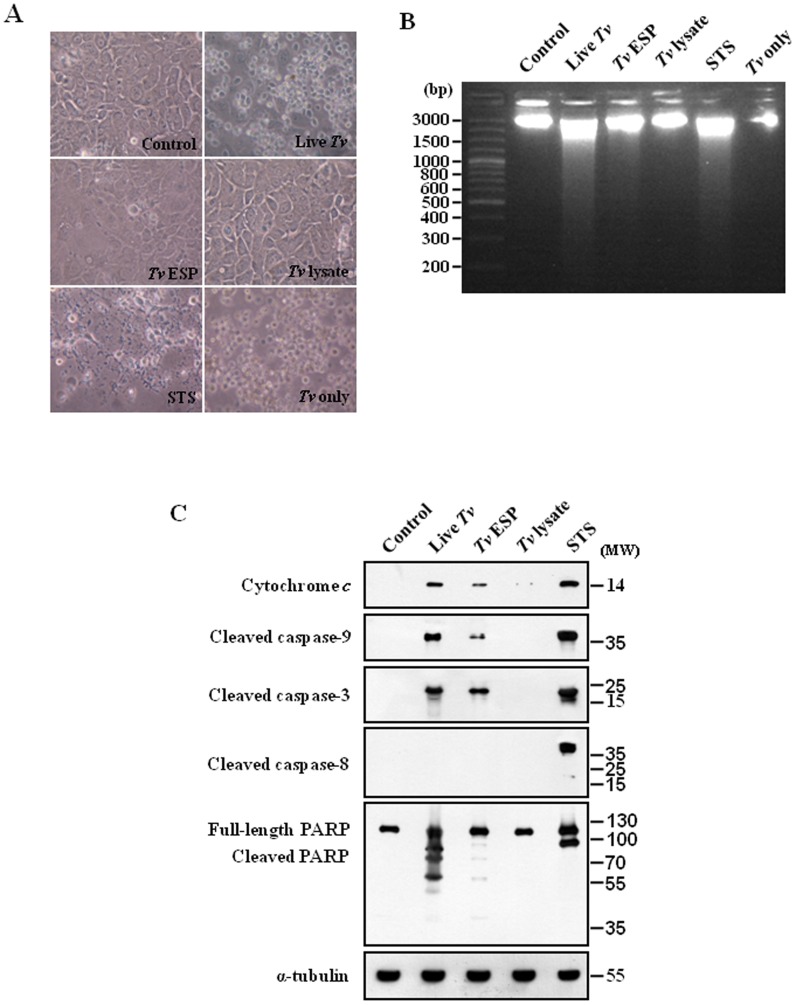
Mitochondria-dependent apoptosis in MS74 cells after treatment with *Trichomonas vaginalis* antigens. (A) Micrographs of MS74 cells were treated as in Fig. 1A. (B) DNA fragmentations of the MS74 cells were determined by agarose-gel electrophoresis. (C) The protein extracts of the MS74 cells were subjected to western blot analysis. Anti-α-tubulin antibodies were used to confirm the equal loading of the cell extracts.

To study how *T. vaginalis* induces cell death in MS74 cells (Fig. 6A), the MS74 cells were treated as in Fig. 1A. As shown in Fig. 6A, treatment of staurosporine, live *T. vaginalis*, or *T. vaginalis* ESP induced the cell death, which was similar to the cases of the SiHa cells (Fig. 1A). The nucleosomal DNA fragmentation was induced in MS74 cells treated with the live *T. vaginalis, T. vaginalis* ESP, or STS, but not by *T. vaginalis* lysate (Fig. 6B). These results suggested that live *T. vaginalis* and ESP from *T. vaginalis* also induce MS74 cell death. In order to assess the apoptotic signaling pathways involved in the *T. vaginalis*-treated MS74 cells, we performed western blotting using apoptosis related antibodies. As shown in Fig. 6C, cytochrome *c* release, cleaved caspase-9, -3, -8 and PARP in the MS74 cells treated with live *T. vaginalis, T. vaginalis* ESP, or *T. vaginalis* lysate were similar to those of SiHa cells (Fig. 1C). Treatment of live *T. vaginalis* or *T. vaginalis* ESP produced apparently cleaved forms of caspase-3 in the MS74 cells, like the staurosporine treatment. In the MS74 cells treated with live *T. vaginalis* or *T. vaginalis* ESP, we observed cleaved form p37 of caspase-9, and also strong cytosolic cytochrome *c*, which were detected also in staurosporine-treated MS74 cells. In contrast, no cleaved forms of caspase-8 was detectable in the live *T. vaginalis*-treated MS74 cells.

**Figure 7:**
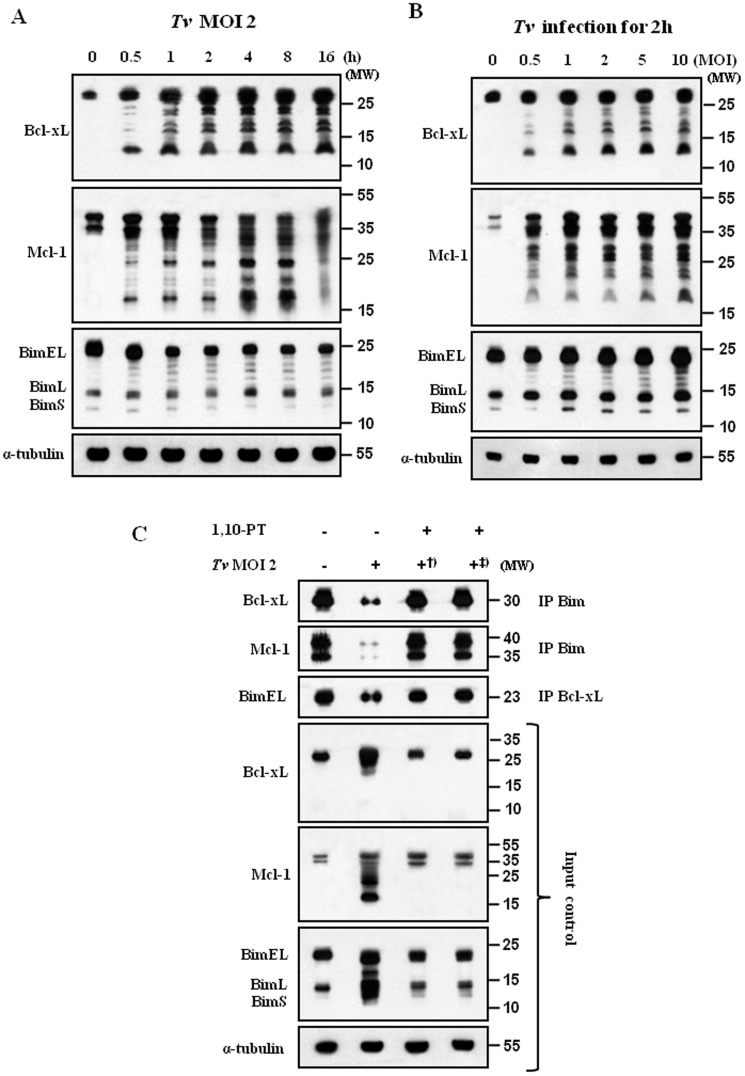
*T. vaginalis* metalloproteinase induces apoptosis of MS74 cells through disrupting the Mcl-1/Bim and Bcl-xL/Bim complexes. MS74 cells were stimulated with *T. vaginalis* (MOI = 2) for the indicated times (A) or at the indicated MOI for 2 h (B). MS74 cell lysates were analyzed by western blotting using indicated antibodies. (C) MS74 cells were treated as Fig. 5B, and whole-cell lysates were subjected to IP using anti-Bim and anti-Bcl-xL antibodies. IP and input samples were resolved by SDS-PAGE and probed with the indicated antibodies.

We examined the changes in the Bcl-2 family members using MS74 cells, treatment of live *T. vaginalis* at MOI 2 produced a strong cleavage of Mcl-1 and Bcl-xL in the MS74 cells at early time of 30 min (Fig. 7A). Similar data were obtained using MS74 cells, infection with *T. vaginalis* resulted in a MOI-dependent cleavage of Bcl-xL and Mcl-1 (Fig. 7B). To confirm that metalloproteinases are indeed critical for the stability of Bcl-xL/Bim and Mcl-1/Bim complexes, we examined the effects of 1,10-PT on the disruption of Bcl-xL/Bim and Mcl-1/Bim complexes in MS74 cells. As expected, we found that treatments of 1,10-PT, prevent the disruption of Bcl-xL/Bim and Mcl-1/Bim complexes in *T. vaginalis*-infected MS74 cells (Fig. 7C). Therefore, we conclude that metalloproteinases are indeed critical for the stability of Bcl-xL/Bim and Mcl-1/Bim complexes, which is important for the mitochondria-dependent apoptotic pathway.

## Discussion

In the present study, we reported the involvement of metalloproteases in *T. vaginalis-*induced cell death in SiHa and MS74 cells. Treatment of 1,10-PT, the inhibitor of zinc-dependent metalloproteases, strongly inhibited the various aspects of *T. vaginalis*-induced cell death such as DNA fragmentation, cleavage of Bcl-xL and Mcl-1, disruption of Bim/Mcl-1 and Bim/Bcl-xL complexes, cytochrome *c* release, caspase-9 and -3 activation, and PARP cleavage. Collectively, our results suggest that the *T. vaginalis* metalloproteinases participated in mitochondria-dependent apoptosis in cervicovaginal cells by inducing the cleavage of Bcl-xL and Mcl-1 as well as the disruption of Bcl-xL/Bim and Mcl-1/Bim complexes.

Our results suggested that *T. vaginalis* GP63-like proteases are critical in the cell death of the SiHa cells in the host-pathogen interaction. *T. vaginalis* encodes several proteases that hydrolyze the mucosal and extracellular matrix proteins of its host. Previous studies suggested that cell surface proteins in the extracellular matrices of the host cells are involved in their interactions with *T. vaginalis* and thus critical to subsequent cell death of the target host cells [9], [13]. The second largest gene family of candidate surface proteins in *T. vaginalis* was known to be GP63-like proteins, most of which contains the minimal motifs HEXXH [24], [25]. GP63 proteases in *Leishmania* were characterized to be zinc metallopeptidase with a zinc-binding motif of HEXXH [24]. The GP63 proteases related to the cleavage of host cell macromolecules [25] were also identified as the major pathogenic agent of *Leishmania* spp. [24] to play an important role in host–parasite interactions [26]. The sequence features of *T. vaginalis* GP63-like proteins and the functional data from other parasites suggest that these proteins are likely to play critical roles in *T. vaginalis* pathogenicity [12]. The 1,10-PT, which worked as a specific inhibitor of the GP63 family in *Leishmania* [25], [27], was chosen as the most favorable reagent for studying the functions of the GP63-like protease family in *T. vaginalis*, since we do not have enough knowledge concerning which kinds of *T. vaginalis* GP63 are effectively involved in the parasite cytotoxicity, and it was known that there are so many members of the *T. vaginalis* GP63-like proteins family. In our study, 1,10-PT treatment almost completely blocked the apoptosis of the target cells induced by live *T. vaginalis* or *T. vaginalis* ESP. A strong band of gelatinase activity around 71 kDa detected in live *T. vaginalis* was almost completely inhibited by 1,10-PT. *T. vaginalis* ESP was also found to have the gelatinase activity band of the same size inhibited by 1,10-PT, suggesting that, in addition to the cell-associated metalloprotease, *T. vaginalis* have the extracellularly released metalloprotease critical for its pathogenicity. It was reported that the copper-1,10-PT complex showed apoptosis-inducing effects on the treated cells [28]. Mechanically, these effects are possibly due to the radical forming effects of copper chelated by 1,10-PT and are not seemingly relevant to our observations. The effects of the copper-1,10-PT complex in the previous study was apoptosis-inducing, very opposite to the anti-apoptotic effects of 1,10-PT in our study, suggesting that their molecular and biochemical basis should be different. A low concentration of 1,10-PT was used, in our study, to avoid its harmful or side effect on host cell and *T. vaginalis*, and was exposed for a short time (30 min) and then washed out clearly. Our results suggest that the action of zinc critical for GP63-like protease activity in *T. vaginalis* would be inhibited by 1,10-PT and, therefore, the host cell death induced by GP63-like protease was prevented.

Here, we reported the identification of a mitochondrial apoptotic pathway for *T. vaginalis*-induced cell death of the SiHa and MS74 cells. Apoptosis is induced by the activation of a series of enzymes known as caspases. The balance among proteolytic enzyme levels, mitochondrial protein localization, the regulation of intracellular signaling, and gene expression is a key factor in deciding cell fates [4]. There were several lines of evidence that *T. vaginalis* employs various strategies for inducing apoptosis in host cells [6]–[8]: *T. vaginalis* induced the apoptosis by activating caspase-3 and reducing Mcl-1 expression [6] and regulated through Bcl-xL, but not Bcl-2 [8]. However, these reports did not provide systematic approaches of apoptosis induced by *T. vaginalis*. Our study presented better understanding of several critical processes involved in the *T. vaginalis*-induced mitochondrial apoptotic pathway; the cleavage of Bcl-xL and Mcl-1, the disruption of the interactions between Bim/Bcl-xL and Bim/Mcl-1, caspase-9 and -3 activation, PARP cleavage, and cytochrome *c* release into cytosol. In this study, we found that *T. vaginalis* treatment led to a rapid and marked cleavage of Mcl-1 and Bcl-xL protein in a dose-dependent way, which was accompanied by an increase in cleavage of caspase-3. Caspase 3-dependent cleavage of Mcl-1 and Bcl-xL was suggested to promote the mitochondria-dependent apoptosis. Previous results showed that *T. vaginalis*-induced apoptosis was associated with reduced expression of Mcl-1 [6] and down-regulation of Bcl-xL [8]. However, they did not explain how their interaction with BH-3 only proteins plays an important role in the apoptotic process induced by *T. vaginalis*.

Our data support a model whereby the disruption of the Mcl-1/Bim and Bcl-xL/Bim complex initiates a Bim-mediated cellular cytotoxic mechanism that requires the initial cleavage of Mcl-1 and Bcl-xL, resulting in the release of mitochondrial Bim from Mcl-1 and Bcl-xL sequestration. The principal role of anti-apoptotic Bcl-xL and Mcl-1 in preventing apoptosis was proposed to sequester the BH3-only molecules such as Bim, tBid, and Puma into stable complexes, thus preventing the activation of Bak and Bax [23], [29], [30]. Bim as the direct activator is not only able to interact with and be sequestered by the anti-apoptotic Bcl-2 proteins Mcl-1 and Bcl-xL but also directly bind to and activate the effectors Bax and Bak [23]. The multidomain pro-apoptotic Bcl-2 proteins Bax and Bak are two major effectors of mitochondrial outer membrane permeabilization, which homo-oligomerize and form pores in the mitochondrial outer membrane to induce mitochondrial outer membrane permeabilization, leading to the release of cytochrome *c* from the mitochondria into the cytosol. The high affinity binding of Mcl-1 with Bim may be at the crux of its anti-apoptotic effect, which is most likely accomplished through the sequestration of this potent pro-apoptotic protein Bim [29]. We found that there is the constitutive binding of Bim with anti-apoptotic proteins Mcl-1 and Bcl-xL, supporting the notion that anti-apoptotic molecules have an important role in neutralization of Bim and thus prevent the activation of death effectors such as Bak and Bax [31]-[33]. Interestingly, a time- and dose-dependent decrease in the protein expression of Bim was also observed following the cleavage of Mcl-1 and Bcl-xL in the cells treated with *T. vaginalis*. Because free Mcl-1 and Bim were reported to be more susceptible to proteasomal degradation [34], the decreases in Bim protein levels in *T. vaginalis*-treated cells are probably due to the disruption of the Bim/Mcl-1 and Bim/BcL-xL complex by *T. vaginalis*. In fact, treatment with *T. vaginalis* disrupted the interaction between Bim and Mcl-1, as demonstrated by co-immunoprecipitation. This study also showed that *T. vaginlis* treatment resulted in reduction of the amount of pro-apoptotic protein Puma, Bid, Bim, Bax and Bak, which were detected at 16 h after treatment with live *T. vaginalis*. However, the anti-apoptotic Bcl-2 proteins Mcl-1 and Bcl-xL started to be cleaved already within 30 min and their anti-apoptotic mechanism were prevented much earlier than the decrease of the amount of pro-apoptotic protein. Pro-apoptotic Bcl-2 family proteins may be cleaved or degraded by proteinases activated in the apoptotic process which was already turned on much earlier. These phenomena were proved at treatment with *T. vaginalis* lysate for a long time or treatment with high dose of *T. vaginalis* for a short time. Treatment with *T. vaginalis* lysate for 16 h did not induce any change in the expressions of pro-apoptotic proteins, and protein expression levels of BimEL, BimL and BimS were remarkably reduced at high burden of *T. vaginalis* (MOI 10) for 2 h. When the host cells were exposed to appropriate number of *T. vaginalis* even for a long time, pro-apoptotic proteins, such as Bim, Bak and Bax, experienced relatively small decrease by *T. vaginalis* infection and their amounts were maintained in certain level, suggesting that they may act as effectors of mitochondrial apoptosis. Collectively, our results suggest that the disruption of the Bim/Mcl-1 and Bim/BcL-xL complex were started early by *T. vaginalis* treatment and then released pro-apoptotic proteins initiated the apoptotic processes.

**Figure 8:**
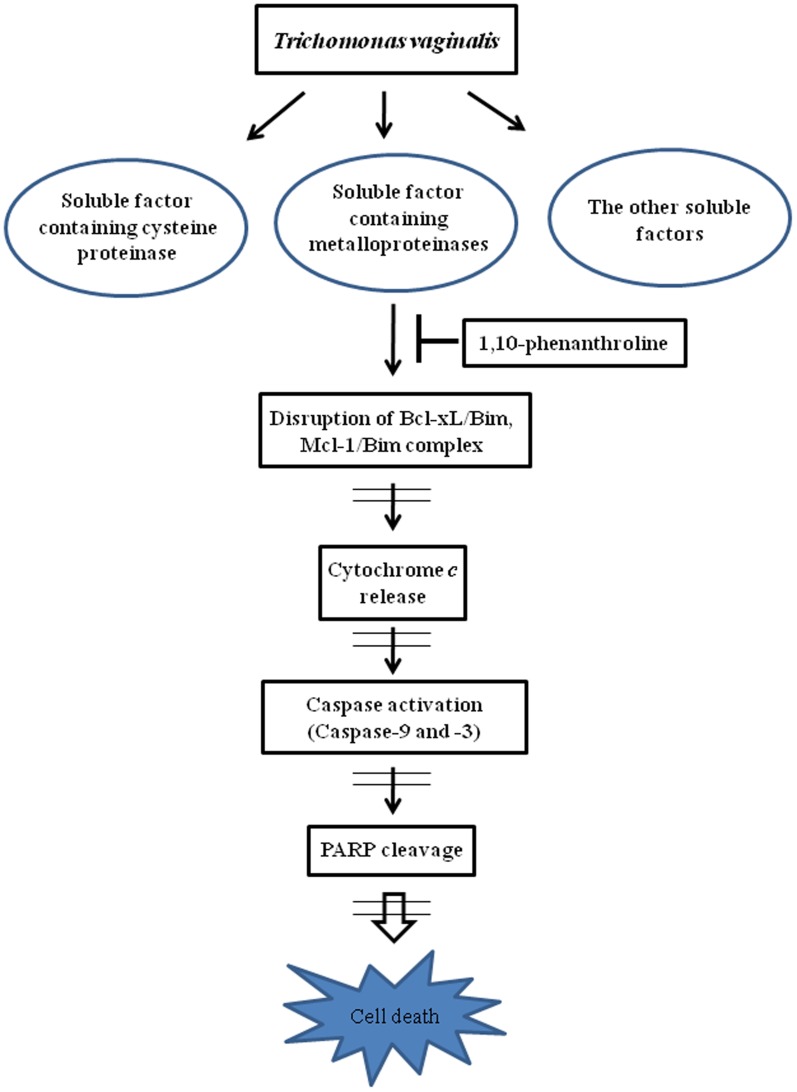
Suggested apoptotic signaling pathway in SiHa cells induced by *T. vaginalis* metalloproteinases. *T. vaginalis* has a number of soluble factors that contribute to pathogenesis. *T. vaginalis* metalloproteinases disrupt the Bcl-xL/Bim and Mcl-1/Bim complexes and then induce apoptosis.

In this study, we reported a critical role of metalloproteases in *T. vaginalis*-induced cell death of the cervical cancer cell line SiHa cells and immortalized vaginal epithelial cell line MS74 cells, even though *T. vaginalis* produces other types of proteases such as cysteine proteases and serine proteases (Fig. 8). The ESP from *T. vaginalis* has the similar effects on the SiHa cells like live *T. vaginalis*. In *T. vaginalis*-induced apoptotic process there was a strong involvement of signaling through the mitochondria such as release of cytochrome *c* from the mitochondria, subsequent caspase-9 cleavage, and activation of caspase-3. As upstream processes of the cytochrome *c* release from the mitochondria, *T. vaginalis* infection induced the rapid cleavage of Bcl-xL and Mcl-1, leading to disruption of Bim/Mcl-1 and Bim/Bcl-xL complexes. The critical function of anti-apoptotic Bcl-xL and Mcl-1 in preventing apoptosis has been proposed to sequester BH3-only molecules into stable complexes, thus preventing the activation of Bax and Bak, and also a BH3-only molecule Bim was shown to directly activate Bax and Bak to release cytochrome *c* from the mitochondria. Treatment of 1,10-PT, the inhibitor of zinc-dependent metalloproteases, strongly inhibited the various aspects of *T. vaginalis*-induced cell death. To our knowledge, this is the first report of the apoptotic activities of *T. vaginalis* metalloproteinases in human cervicovaginal cells and of the mechanisms by which metalloproteinases exert their effects on the Bcl-2 family proteins. Since *T. vaginalis* metallopeptidases released onto the host mucosal surface is an important pathogenetic factor, thus this study provides a new understanding of the regulatory role of metalloproteinases activity and mitochondrial apoptosis-signaling pathways in trichomoniasis of the cervicovaginal epithelial cells.

## Supporting Information

File S1 Figure S1, Analysis of caspase-3 cleavage in SiHa cells treated with various amounts of *T. vaginalis* antigens. The optimal incubation time for inducing apoptosis was 16 h. The optimal concentrations of live *T. vaginalis, T. vaginalis* excretory and secretory products (ESP), and *T. vaginalis* lysate for inducing apoptosis were an MOI of 2, 100 µg/mL, and 100 µg/mL, respectively. Figure S2, Viability of *T. vaginalis* cells after treatment with 1,10-PT. Live *T. vaginalis* cells were treated with metalloproteinase inhibitor 1,10-phenanthroline (1,10-PT) for 30 min, and viability was assayed using trypan-blue staining. *T. vaginalis* viability was decreased by the application of ≥7 mM 1, 10-PT. Figure S3, Effects of protease activity after treatment with inhibitors. At Siha cell monolayer, *T. vaginalis* (MOI = 2) was treated for 30 min with 1,10-PT and caspase-3 inhibitor Z-DEVD-FMK at 37°C. The supernatant was collected, and protease activity was determined by casein hydrolysis assay. * *P*<0.05 compared with the control group (no inhibitor). Figure S4, Comparison of the effects of 1,10-PT and Z-DEVD-FMK on the *T. vaginalis*-induced cleavage of PARP, Mcl-1, Bcl-xL and Bim. The caspase-3 inhibitor Z-DEVD-FMK slightly suppressed apoptosis. The metalloproteinase inhibitor 1,10-PT strongly suppressed apoptosis. Both caspase-3 and *T. vaginalis* metalloproteinases are involved in the cleavage of Bcl-xL and Mcl-1 and the degradation of Bim; however, the *T. vaginalis* metalloproteinases are more potent for inducing the cleavage or degradation of the Bcl-2 proteins.
(PPT)

## References

[1] Van der Pol B (2007) *Trichomonas vaginalis* infection: the most prevalent nonviral sexually transmitted infection receives the least public health attention. Clin Infect Dis 44: 23–25. 17143810 10.1086/509934

[2] Soper D (2004) Trichomoniasis: under control or undercontrolled? Am J Obst Gyn 190: 281–290. 10.1016/j.ajog.2003.08.023 14749674

[3] Sinha K, Das J, Pal PB, Sil PC (2013) Oxidative stress: the mitochondria-dependent and mitochondria-independent pathways of apoptosis. Arch Toxicol 87: 1157–1180. 23543009 10.1007/s00204-013-1034-4

[4] Zimmermann KC, Green DR (2001) How cells die: apoptosis pathways. J Allergy Clin Immunol 108: S99–S103. 11586274 10.1067/mai.2001.117819

[5] Youle RJ, Strasser A (2008) The BCL-2 protein family: opposing activities that mediate cell death. Nat Rev Mol Cell Biol 9: 47–59. 18097445 10.1038/nrm2308

[6] Kang JH, Song HO, Ryu JS, Shin MH, Kim JM, et al (2006) *Trichomonas vaginalis* promotes apoptosis of human neutrophils by activating caspase-3 and reducing Mcl-1 expression. Parasite Immunol 28: 439–446. 16916367 10.1111/j.1365-3024.2006.00884.x PMC2562650

[7] Song HO, Shin MH, Ahn MH, Min DY, Kim YS, et al (2008) *Trichomonas vaginalis*: Reactive oxygen species mediates caspase-3 dependent apoptosis of human neutrophils. Exp Parasitol 118: 59–65. 17709105 10.1016/j.exppara.2007.06.010

[8] Chang JH, Ryang YS, Kim SK, Park JY (2004) *Trichomonas vaginalis*-induced apoptosis in RAW264.7 cells is regulated through Bcl-xL, but not Bcl-2. Parasite Immunol 26: 141–150. 15279625 10.1111/j.0141-9838.2004.00693.x

[9] Figueroa-Angulo EE, Rendón-Gandarilla FJ, Puente-Rivera J, Calla-Choque JS, Cárdenas-Guerra RE, et al (2012) The effects of environmental factors on the virulence of *Trichomonas vaginalis* . Microbes Infect 14: 1411–1427. 23022315 10.1016/j.micinf.2012.09.004

[10] Ryan CM, de Miguel N, Johnson PJ (2011) *Trichomonas vaginalis*: current understanding of host-parasite interactions. Essays Biochem 51: 161–175. 22023448 10.1042/bse0510161 PMC6445371

[11] Sommer U, Costello CE, Hayes GR, Beach DH, Gilbert RO, et al (2005) Identification of *Trichomonas vaginalis* cysteine proteases that induce apoptosis in human vaginal epithelial cells. J Biol Chem 280: 2353–2360. 10.1074/jbc.M501752200 15843376

[12] Ma L, Meng Q, Cheng W, Sung Y, Tang P, et al (2011) Involvement of the GP63 protease in infection of *Trichomonas vaginalis* . Parasitol Res 109: 71–79. 21221643 10.1007/s00436-010-2222-2

[13] Hirt RP, Noel CJ, Sicheritz-Ponten T, Tachezy J, Fiori PL (2007) *Trichomonas vaginalis* surface proteins: a view from the genome. Trends Parasitol 23: 540–547. 17962075 10.1016/j.pt.2007.08.020

[14] Felber JP, Coombs TL, Vallee BL (1962) The mechanism of inhibition of carboxypeptidase A by 1,10-phenanthroline. Biochemistry 1: 231–238. 13892106 10.1021/bi00908a006

[15] Correa LM, Cho C, Myles DG, Primakoff P (2000) A role for a TIMP-3-sensitive, Zn(2+)-dependent metalloprotease in mammalian gamete membrane fusion. Dev Biol 225: 124–134. 10964469 10.1006/dbio.2000.9825

[16] Musatovova O, Alderete JF (1999) The *Trichomonas vaginalis* phenotypically varying P270 immunogen is highly conserved except for numbers of repeated elements. Microb Pathog 27: 93–104. 10458920 10.1006/mpat.1999.0281

[17] Song HO, Lim YS, Moon SJ, Ahn MH, Ryu JS (2010) Delayed human neutrophil apoptosis by *Trichomonas vaginalis* lysate. Korean J Parasitol 48: 1–7. 20333279 10.3347/kjp.2010.48.1.1 PMC2843841

[18] Han IH, Park SJ, Ahn MH, Ryu JS (2012) Involvement of mast cells in inflammation induced by *Trichomonas vaginalis* via crosstalk with vaginal epithelial cells. Parasite Immunol 34: 8–14. 21981317 10.1111/j.1365-3024.2011.01338.x

[19] Kucknoor A, Mundodi V, Alderete JF (2005) *Trichomonas vaginalis* adherence mediates differential gene expression in human vaginal epithelial cells. Cell Microbiol 7: 887–897. 15888089 10.1111/j.1462-5822.2005.00522.x PMC2562669

[20] Quan JH, Cha GH, Zhou W, Chu JQ, Nishikawa Y, et al (2013) Involvement of PI 3 kinase/Akt-dependent Bad phosphorylation in *Toxoplasma gondii*-mediated inhibition of host cell apoptosis. Exp Parasitol 133: 462–471. 23333591 10.1016/j.exppara.2013.01.005

[21] Fujita N, Nagahashi A, Nagashima K, Rokudai S, Tsuruo T (1998) Acceleration of apoptotic cell death after the cleavage of Bcl-xL protein by caspase-3-like proteases. Oncogene 17: 1295–1304. 9771973 10.1038/sj.onc.1202065

[22] Weng C, Li Y, Xu D, Shi Y, Tang H (2005) Specific Cleavage of Mcl-1 by caspase-3 in tumor necrosis factor-related aApoptosis-inducing ligand (TRAIL)-induced apoptosis in Jurkat Leukemia T Cells. J Biol Chem 280: 10491–10500. 15637055 10.1074/jbc.M412819200

[23] Kim H, Rafiuddin-Shah M, Tu HC, Jeffers JR, Zambetti GP, et al (2006) Hierarchical regulation of mitochondrion-dependent apoptosis by BCL-2 subfamilies. Nat Cell Biol 8: 1348–1358. 17115033 10.1038/ncb1499

[24] Joshi PB, Kelly BL, Kamhawi S, Sacks DL, McMaster WR (2002) Targeted gene deletion in *Leishmania major* identifies leishmanolysin (GP63) as a virulence factor. Mol Biochem Parasitol 120: 33–40. 11849703 10.1016/s0166-6851(01)00432-7

[25] Chaudhuri G, Chaudhuri M, Pan A, Chang KP (1989) Surface acid proteinase (gp63) of *Leishmania mexicana*. A metalloenzyme capable of protecting liposome-encapsulated proteins from phagolysosomal degradation by macrophages. J Biol Chem 264: 7483–7489. 2708373

[26] Bouvier J, Schneider P, Etges R (1995) Leishmanolysin: surface metalloproteinase *of Leishmania* . Methods Enzymol 248: 614–633. 7674949 10.1016/0076-6879(95)48039-0

[27] Seay MB, Heard PL, Chaudhuri G (1996) Surface Zn-proteinase as a molecule for defense of *Leishmania mexicana* amazonensis promastigotes against cytolysis inside macrophage phagolysosomes. Infect Immun 64: 5129–5137. 8945556 10.1128/iai.64.12.5129-5137.1996 PMC174498

[28] Zhang Z1, Bi C, Schmitt SM, Fan Y, Dong L, et al (2012) 1,10-Phenanthroline promotes copper complexes into tumor cells and induces apoptosis by inhibiting the proteasome activity. J Biol Inorg Chem 17: 1257–1267. 23053530 10.1007/s00775-012-0940-x PMC3662054

[29] Han J1, Goldstein LA, Gastman BR, Froelich CJ, Yin XM, et al (2004) Degradation of Mcl-1 by granzyme B: implications for Bim-mediated mitochondrial apoptotic events. J Biol Chem 279: 22020–22029. 15014070 10.1074/jbc.M313234200

[30] Opferman JT, Letai A, Beard C, Sorcinelli MD, Ong CC, et al (2003) Development and maintenance of B and T lymphocytes requires antiapoptotic MCL-1. Nature 426: 671–676. 14668867 10.1038/nature02067

[31] Opferman JT, Korsmeyer SJ (2003) Apoptosis in the development and maintenance of the immune system. Nat Immunol 4: 410–415. 12719730 10.1038/ni0503-410

[32] Cheng EH, Wei MC, Weiler S, Flavell RA, Mak TW, et al (2001) Bcl-2, Bcl-x(L) sequester BH3 domain-only molecules preventing Bax- and Bak-mediated mitochondrial apoptosis. Mol Cell 8: 705–711. 11583631 10.1016/s1097-2765(01)00320-3

[33] Zong WX, Lindsten T, Ross AJ, MacGregor GR, Thompson CB (2001) BH3-only proteins that bind pro-survival Bcl-2 family members fail to induce apoptosis in the absence of Bax and Bak. Genes Dev 15: 1481–1486. 11410528 10.1101/gad.897601 PMC312722

[34] Wuillème-Toumi S, Trichet V, Gomez-Bougie P, Gratas C, Bataille R, et al (2007) Reciprocal protection of Mcl-1 and Bim from ubiquitin-proteasome degradation. Biochem Biophys Res Commun 361: 865–869. 17681275 10.1016/j.bbrc.2007.07.070

