# Supplementary material for: Trichonomas vaginalis Metalloproteinase Induces Apoptosis of SiHa Cells through Disrupting the Mcl-1/Bim and Bcl-xL/Bim Complexes
Source: PLoS One. 2014 Oct 24;9(10):e110659. doi: 10.1371/journal.pone.0110659 (PMC4208800; doi:10.1371/journal.pone.0110659)
Supplement: File S1 — Figure S1, Analysis of caspase-3 cleavage in SiHa cells treated with various amounts of T. vaginalis antigens. The optimal incubation time for inducing apoptosis was 16 h. The optimal concentrations of live T. vaginalis, T. vaginalis excretory and secretory products (ESP), and T. vaginalis lysate for inducing apoptosis were an MOI of 2, 100 µg/mL, and 100 µg/mL, respectively. Figure S2, Viability of T. vaginalis cells after treatment with 1,10-PT. Live T. vaginalis cells were treated with metalloproteinase inhibitor 1,10-phenanthroline (1,10-PT) for 30 min, and viability was assayed using trypan-blue staining. T. vaginalis viability was decreased by the application of ≥7 mM 1, 10-PT. Figure S3, Effects of protease activity after treatment with inhibitors. At Siha cell monolayer, T. vaginalis (MOI = 2) was treated for 30 min with 1,10-PT and caspase-3 inhibitor Z-DEVD-FMK at 37°C. The supernatant was collected, and protease activity was determined by casein hydrolysis assay. * P<0.05 compared with the control group (no inhibitor). Figure S4, Comparison of the effects of 1,10-PT and Z-DEVD-FMK on the T. vaginalis-induced cleavage of PARP, Mcl-1, Bcl-xL and Bim. The caspase-3 inhibitor Z-DEVD-FMK slightly suppressed apoptosis. The metalloproteinase inhibitor 1,10-PT strongly suppressed apoptosis. Both caspase-3 and T. vaginalis metalloproteinases are involved in the cleavage of Bcl-xL and Mcl-1 and the degradation of Bim; however, the T. vaginalis metalloproteinases are more potent for inducing the cleavage or degradation of the Bcl-2 proteins. (PPT) [file pone.0110659.s001.ppt]

## Slide 1
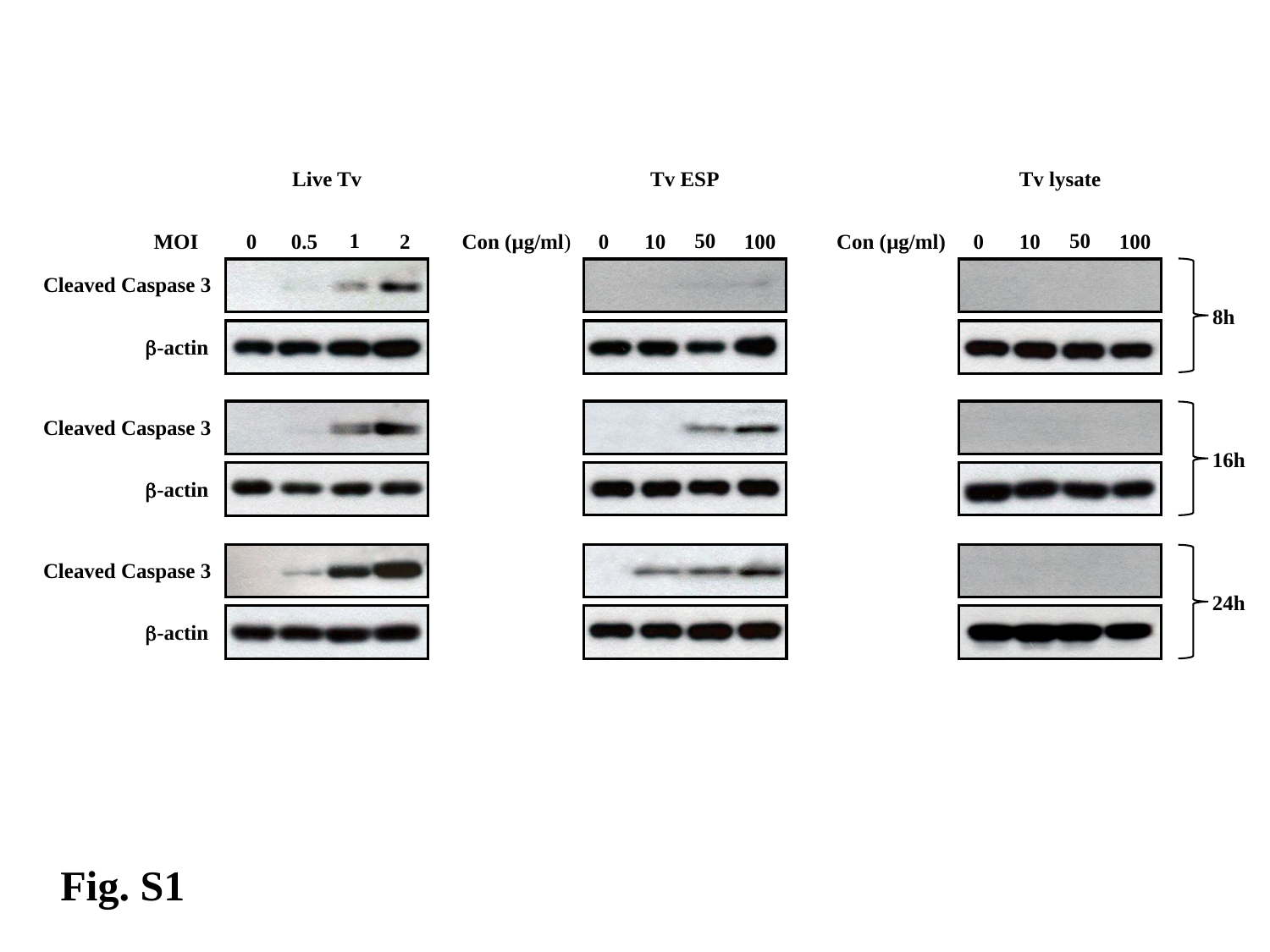

Live Tv
Tv ESP
Tv lysate
| MOI | 0 | 0.5 | 1 | 2 |
| --- | --- | --- | --- | --- |
| Con (μg/ml) | 0 | 10 | 50 | 100 |
| --- | --- | --- | --- | --- |
| Con (μg/ml) | 0 | 10 | 50 | 100 |
| --- | --- | --- | --- | --- |
8h
Cleaved Caspase 3
β-actin
16h
Cleaved Caspase 3
β-actin
24h
Cleaved Caspase 3
β-actin
Fig. S1

## Slide 2
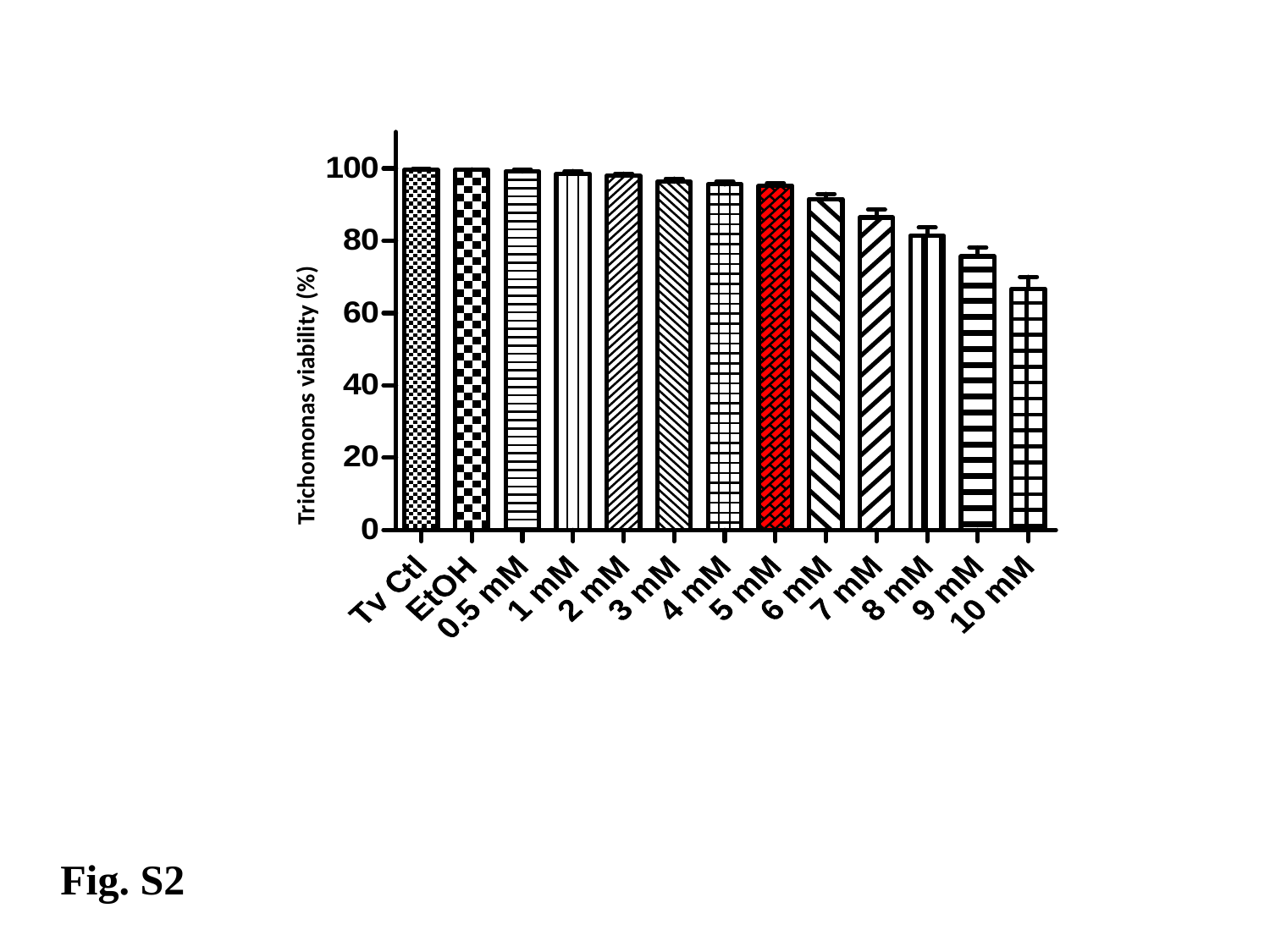

Trichomonas viability (%)
Fig. S2

## Slide 3
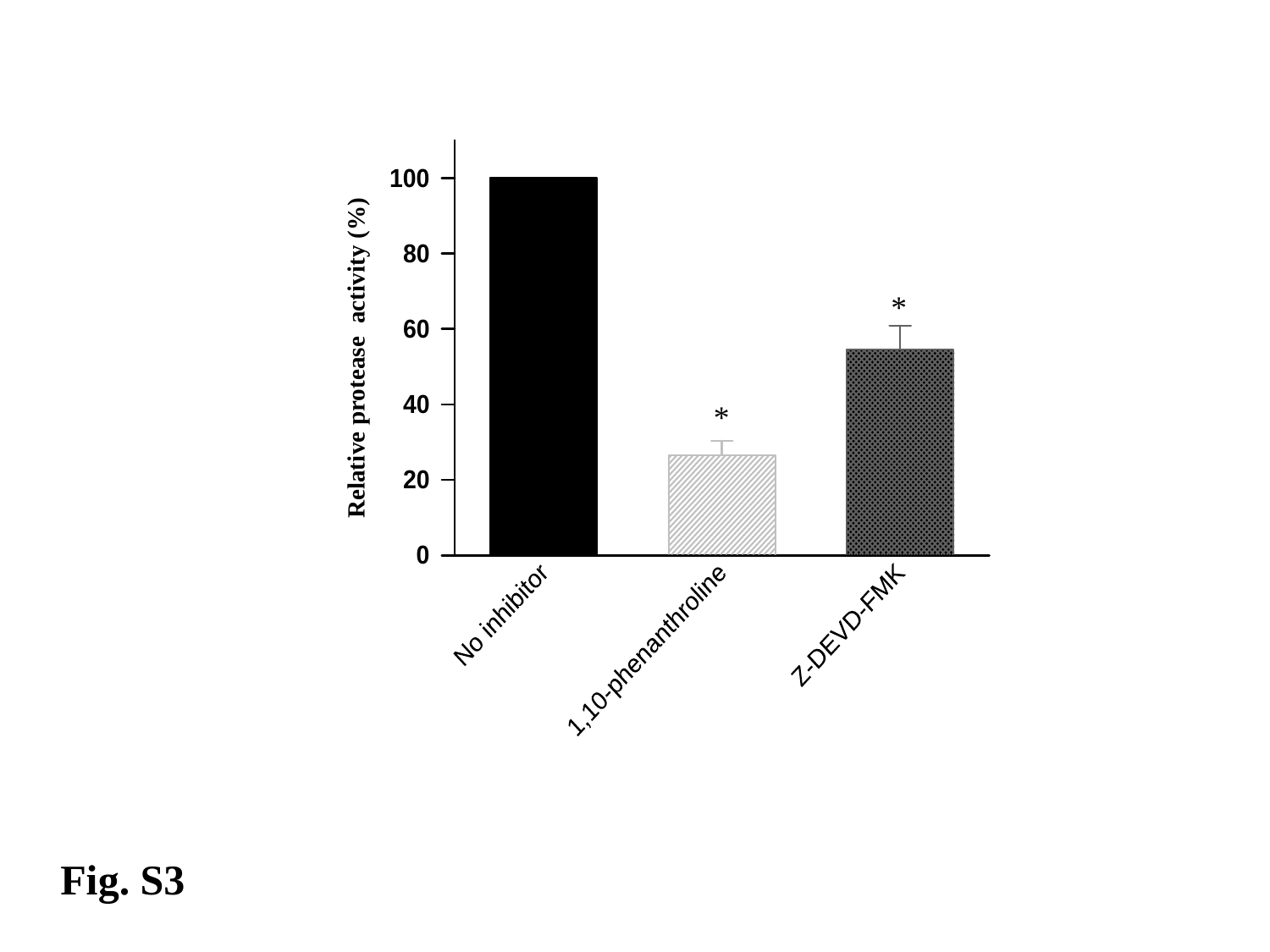

Relative protease activity (%)
*
*
*
Fig. S3

## Slide 4
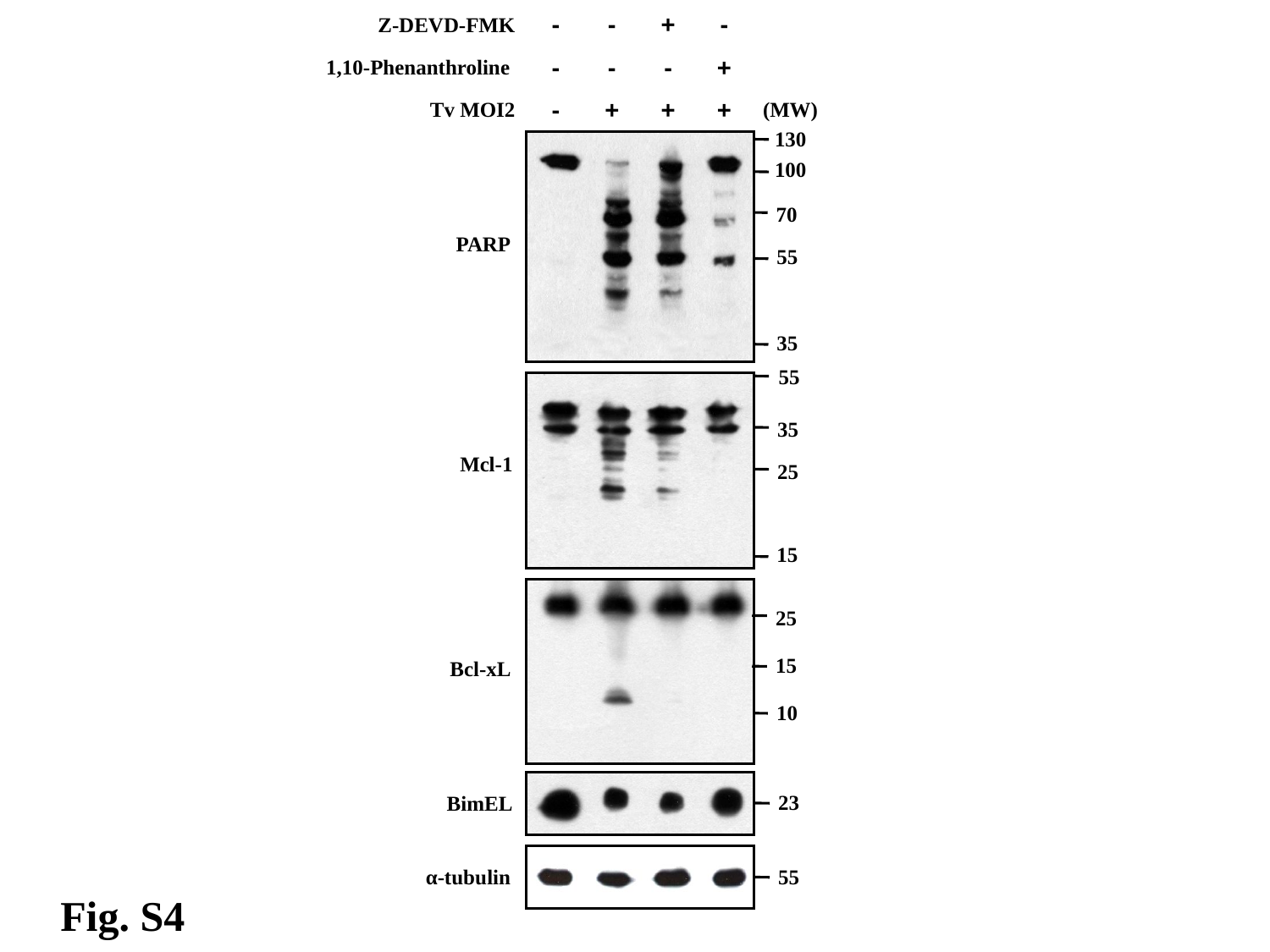

| Z-DEVD-FMK | - | - | + | - |
| --- | --- | --- | --- | --- |
| 1,10-Phenanthroline | - | - | - | + |
| Tv MOI2 | - | + | + | + |
(MW)
130
100
70
55
35
PARP
55
35
Mcl-1
25
15
25
15
Bcl-xL
10
BimEL
23
α-tubulin
55
Fig. S4
